# Anthocyanin regulatory networks in *Solanum tuberosum* L. leaves elucidated via integrated metabolomics, transcriptomics, and *StAN1* overexpression

**DOI:** 10.1186/s12870-022-03557-1

**Published:** 2022-05-04

**Authors:** Yanru Bao, Tengkun Nie, Dongdong Wang, Qin Chen

**Affiliations:** 1 State Key Laboratory of Crop Stress Biology for Arid Areas, College of Food Science and Engineering, Northwest A & F University, Yangling, 712100 Shaanxi China; 2 State Key Laboratory of Crop Stress Biology for Arid Areas, College of Agronomy, Northwest A & F University, Yangling, 712100 Shaanxi China

**Keywords:** Potato, Anthocyanin, Nanopore full-length transcriptome sequencing, Extensive targeted metabolomics, *StAN1*

## Abstract

**Background:**

Anthocyanins, which account for color variation and remove reactive oxygen species, are widely synthesized in plant tissues and organs. Using targeted metabolomics and nanopore full-length transcriptomics, including differential gene expression analysis, we aimed to reveal potato leaf anthocyanin biosynthetic pathways in different colored potato varieties.

**Results:**

Metabolomics analysis revealed 17 anthocyanins. Their levels varied significantly between the different colored varieties, explaining the leaf color differences. The leaves of the Purple Rose2 (PurpleR2) variety contained more petunidin 3-O-glucoside and malvidin 3-O-glucoside than the leaves of other varieties, whereas leaves of Red Rose3 (RedR3) contained more pelargonidin 3-O-glucoside than the leaves of other varieties. In total, 114 genes with significantly different expression were identified in the leaves of the three potato varieties. These included structural anthocyanin synthesis–regulating genes such as *F3H*, *CHS*, *CHI*, *DFR*, and anthocyanidin synthase and transcription factors belonging to multiple families such as C3H, MYB, ERF, NAC, bHLH, and WRKY. We selected an MYB family transcription factor to construct overexpression tobacco plants; overexpression of this factor promoted anthocyanin accumulation, turning the leaves purple and increasing their malvidin 3-o-glucoside and petunidin 3-o-glucoside content.

**Conclusions:**

This study elucidates the effects of anthocyanin-related metabolites on potato leaves and identifies anthocyanin metabolic network candidate genes.

**Supplementary Information:**

The online version contains supplementary material available at 10.1186/s12870-022-03557-1.

## Background

Anthocyanins are important antioxidant flavonoids. In potatoes, they are synthesized by the tubers, stems, leaves, and flowers [1], and can be transported from the aerial parts to the tubers for storage [2–4]. Anthocyanins are responsible for color variation in colored potatoes, which produce both flavonoids and polyphenols [5]. In some varieties, such as Red Rose3 and the Purple Rose2 (The Northwest Agriculture and Forestry University provided the experimental plant materials, hereafter “RedR3” and “PurpleR2”), they cause the tubers and leaves to have the same color. Potatoes, grown in many countries and regions, exhibiting strong adaptability and high yield [6, 7]. As an important food crop, they provide both energy and antioxidants such as ascorbic acid and polyphenols [8]. Further, anthocyanins inhibit aging and prevent cancer [9].

Although leaves play an important role in potato anthocyanin synthesis and accumulation, most research into this has focused on the tubers. Potato plants receive light primarily via their leaves. Anthocyanins exert a protective effect on leaves under biotic and abiotic stress and can heal burns caused by visible and ultraviolet light [10, 11]. Anthocyanin biosynthesis is regulated by transcription factors and related genes that code for enzymes [12, 13].

The oligomerase phenylalanine ammonia lyase (PAL), which links primary and phenylpropanol metabolism in plants, catalyzes the first reaction of phenylalanine metabolism [14, 15]. PAL deaminates phenylalanine, generating trans-cinnamate, which produces cinnamoyl-CoA under the action of 4-coumarate-CoA ligase (4CL); cinnamoyl-CoA is catalyzed by trans-cinnamate 4-monooxygenase (CYP73A) to produce p-coumaroyl-CoA, which ultimately participates in flavonoid biosynthesis [16]. As a catalyst, chalcone synthase (CHS) causes compounds including chalcone isomerase (CHI), naringenin 3-dioxygenase (F3H) [17, 18], and p-coumarinyl-CoA to be converted into anthocyanin precursors such as dihydrokaempferol. Dihydrokaempferol is a key precursor of pelargonidin [19], which is converted to dihydroquercetin under the catalytic action of the flavonoids 3′,5′-hydroxylase (F3′5′H) and 3′-monooxygenase (CYP75B1). Dihydroquercetin is an important precursor of cyanidin [19, 20] that is then catalyzed by F3′5′H to produce dihydromyricetin, an important delphinidin precursor [21, 22]. Dihydroflavonol 4-reductase (DFR) and anthocyanidin synthase (ANS) catalyze the conversion of dihydrokaempferol [23, 24], dihydroquercetin, and dihydromyricetin to the corresponding anthocyanin types. Following its synthesis, anthocyanin accumulates mostly in plant cell vacuoles, primarily as glycosides [25].

Most anthocyanin biosynthesis genes are regulated by the MBW transcription factor complex comprising MYB, bHLH, and WD40 [26, 27]. Transcription factors can activate structural-gene expression. Some early biosynthesis genes are regulated by R2R3-MYB transcription factors; late biosynthesis genes are regulated by other transcription factors [28–30]. In chrysanthemum, a transcription factor of R2R3-MYB directly inhibits DFR gene expression by binding to the promoter of DFR gene [28]. Eggplant’s study also found that transcription factors in this family bind to the CHS promoter and activate its expression [29]. An R2R3-MYB transcription factor SsMYB1 activated anthocyanin biosynthesis by directly binding to the promoters of SsDFR1 and SsANS and promoted their transcription activity in Chinese tallow [30].

We used metabolomics and transcriptomics analyses to elucidate anthocyanin synthesis, regulation, and accumulation in the leaves of different colored potato varieties. These findings aim to provide a theoretical and practical basis to advance research into anthocyanin synthesis and metabolic regulation in potatoes.

## Results

### Leaf anthocyanin leavels

**Fig. 1:**
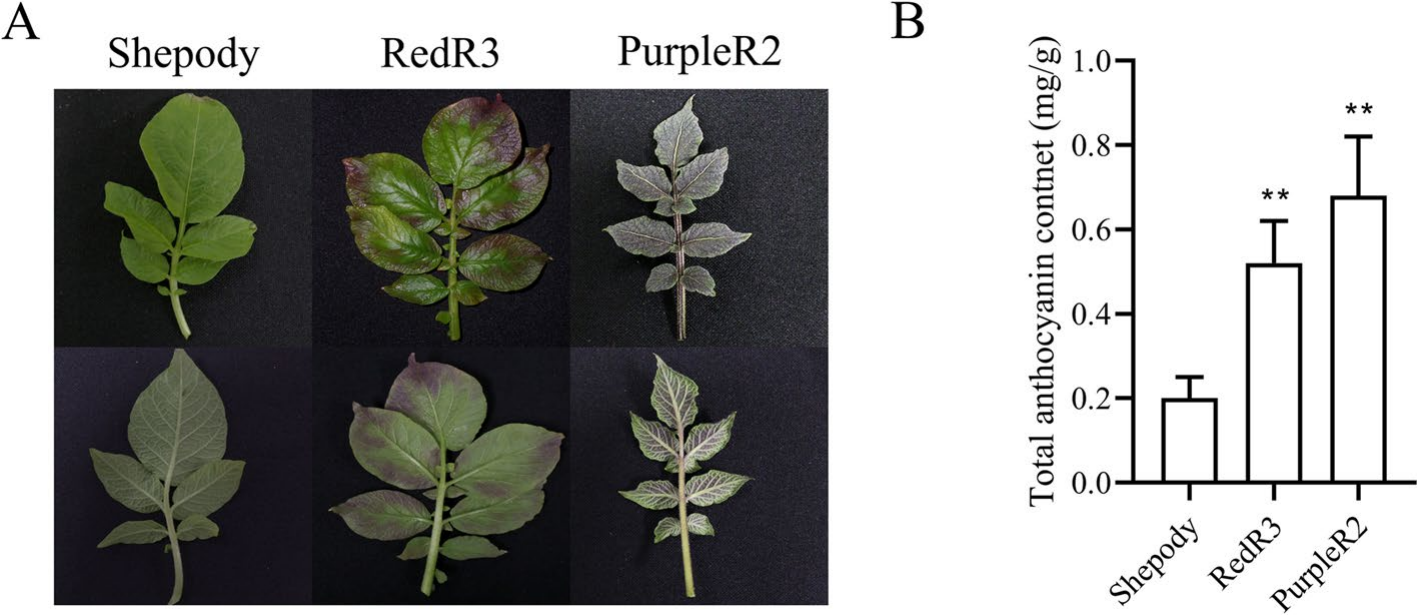
The anthocyanin content of different potato leaves. **A**: The leaves of different potato varieties. **B**: Anthocyanin content in potato leaves of different varieties

Leaf anthocyanin content was 0.52 mg/g in RedR3 and 0.68 in PurpleR2, higher than that in the control (Shepody) (Fig. 1A, B).

### Differential metabolites

**Fig. 2:**
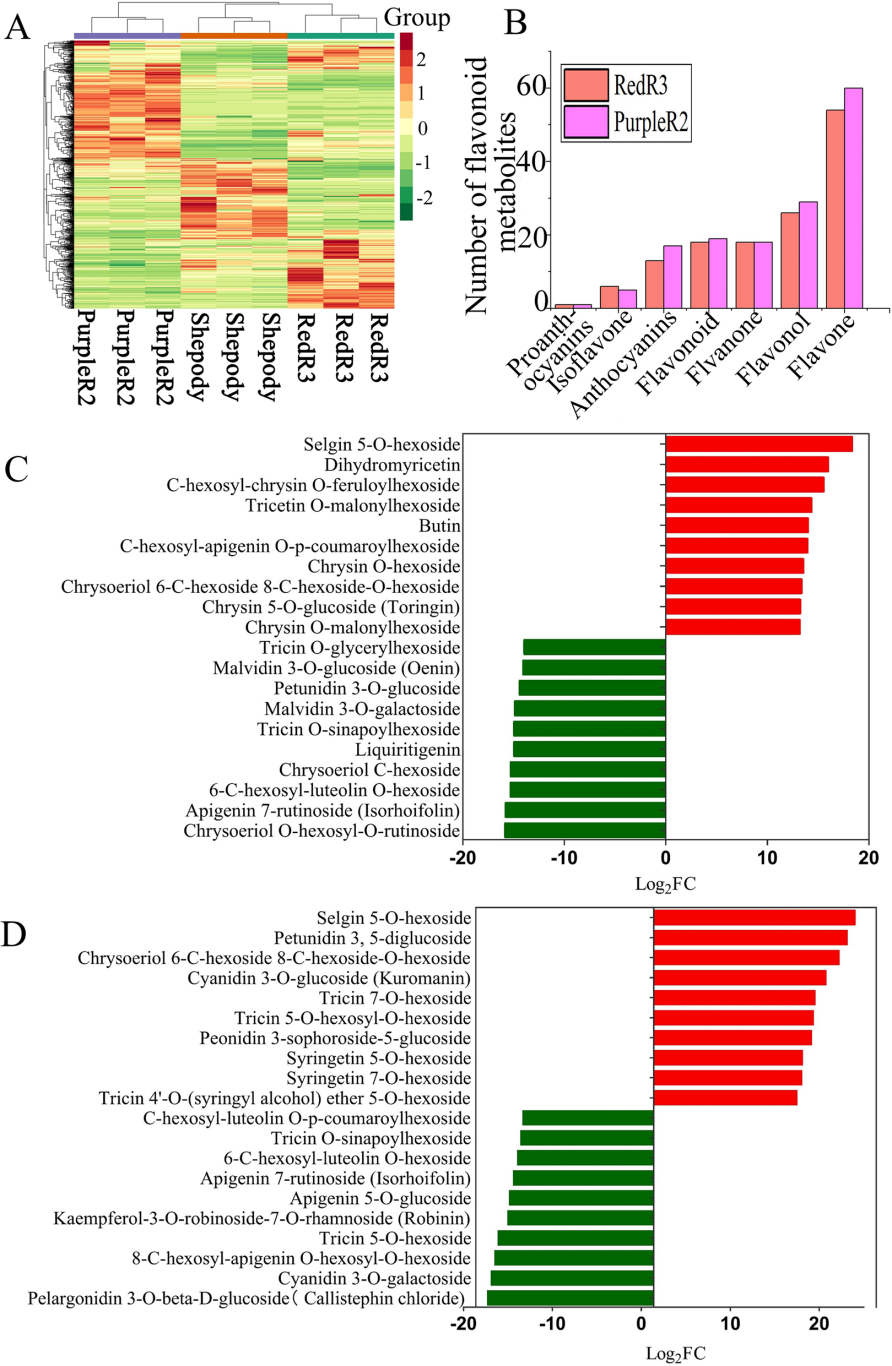
Metabolites in the RedR3 and PurpleR2 potato leaves. **A**: Heatmap of different metabolites. **B**: Distribution of flavonoids in colored potatoes. **C**: Top 20 metabolites in RedR3 potato leaves. **D**: Top 20 metabolites in PurpleR2 potato leaves

We detected 758 metabolites (Table S1), normalized their levels, and generated a heatmap (Fig. 2A). The clustering in the heatmap reveals significant differences in flavonoids between the varieties, with four main clusters. The metabolites in clusters 1 and 4 were most abundant in RedR3, those in cluster 2 were most abundant in PurpleR2, and those in cluster 3 were most abundant in Shepody and relatively scarce in the colored varieties. For each sample, the three biological replicates clustered together, indicating that the biological replicates had good homogeneity and provided reliable data. Differences in flavonoid metabolite content were closely related to leaf color. Relative to those detected in Shepody, 346 and 362 metabolites were detected in RedR3 and PurpleR2, respectively. More than 130 flavonoid metabolites, including apigenin, chrysin, hesperetin, naringenin, luteolin, and their glycosides, were detected (Fig. 2B). Of the anthocyanins, 13 were detected in RedR3, with the contents of cyanidin, delphinidin, pelargonin, and their corresponding glycosides being significantly increased; 17 were detected in PurpleR2, with the contents of cyanidin, malvidin, peonidin, petunidin, and their corresponding glycosides being significantly increased. The top 20 most significantly differentially expressed metabolites (based on |Log2 FC| ≥ 1 and variable importance in projection [VIP] > 1) are shown in Fig. 2. Selgin 5-O-hexoside content was significantly increased in the colored varieties. Among the anthocyanin metabolites, the contents of malvidin 3-O-galactoside, petunidin 3-O-glucoside, and malvidin 3-O-glucoside (oenin) were significantly decreased in RedR3 (Fig. 2C); in PurpleR2, the contents of pelargonidin 3-O-beta-D-glucoside (callistephin chloride) and cyanidin 3-O-galactoside were significantly decreased, whereas those of peonidin 3-sophoroside-5-glucoside, cyanidin 3-O-glucoside (kuromanin), and petunidin 3, 5-diglucoside were significantly increased (Fig. 2D).

### Full-length transcriptome sequencing

**Fig. 3:**
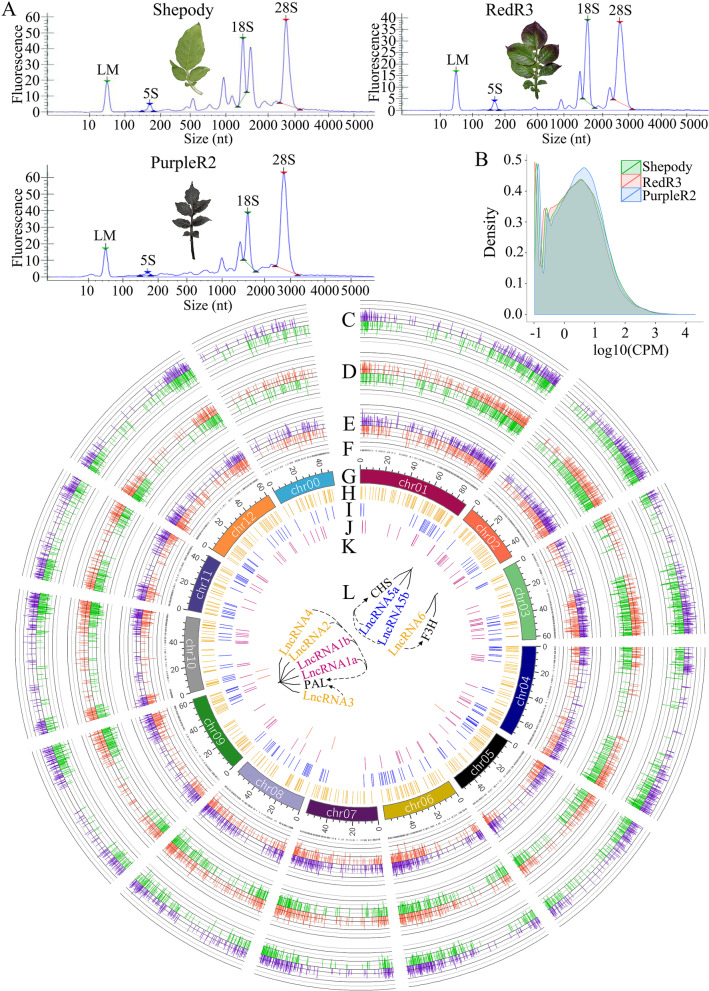
Differential expression of genes in potato leaves of different colors. **A**: Extracted total RNA from potato leaves of different color. **B**: Overall distribution of expressed genes in potato leaves of different colors. **C**: Differentially expressed genes in PurpleR2 were analyzed with Shepody as the control. The purple color represents the up-regulated gene, while green represents the downregulated gene. **D**: The differentially expressed genes in RedR3 were analyzed with Shepody as the control. The red color represents the up-regulated gene, while green represents the downregulated gene. **E**: Comparison of differentially expressed genes between RedR3 and PurpleR2 with RedR3 as a control. Purple color denotes the up-regulated gene, while red denotes the downregulated gene. **F**: Supplementing the annotation information of the potato genome, the distribution of the newly annotated 3543 gene loci on the potato chromosome. **G**: The drawing unit for the Potato genome chromosome was Mb. However, the potato chrUn chromosome is not presented because the sequence could not be matched on the reference genome of known chromosomes. **H**: LincRNA is located on the potato chromosome. **I**: Sense lncRNA is located on the potato chromosome. **J**: Anti-sense lncRNA is located on the potato chromosome. **K**: Intronic lncRNA is located on the potato chromosome. **L**: LncRNA interaction with genes involved in anthocyanin biosynthesis

To explore the molecular basis of flavonoid synthesis in the colored variety leaves, we analyzed the leaf transcriptome via RNA-seq to identify differentially expressed genes (DEGs), and conducted nanopore transcriptome sequencing (RNA sequence integrity results shown in Fig. 3A). Leaves from the three varieties were subjected to full-length transcriptome sequencing, each generating 7.94 Gb of clean data. We combined the full-length transcriptome sequencing data for the samples and removed redundancy after comparison with the reference genome, obtaining 43,575 full-length potato transcript sequences. Shepody and RedR3 had similar gene expression patterns (Fig. 3B).

Pairwise comparison of samples (Fig. 3C–E, Table S2) revealed that the DEGs were distributed on all chromosomes, with many occurring on chromosome 1. Relative to those in Shepody (the control), PurpleR2 had 6145 significantly differentially expressed transcripts (2949 upregulated and 3196 downregulated), and RedR3 had 5789 significantly differentially expressed transcripts (2819 upregulated and 2970 downregulated). Relative to those in RedR3, PurpleR2 had 4947 significantly differentially expressed transcripts (2694 upregulated and 2253 downregulated). The number of differentially expressed genes was found to be similar between the colored varieties compared to the control cultivars, revealing differences in gene expression between the different varieties.

The limitations of second-generation high-throughput sequencing technology prevented us from obtaining sufficiently accurate reference genome annotations. Therefore, to optimize the original genome annotations, we used nanopore full-length transcriptome sequencing, which can accurately identify transcript structures. This revealed 3543 additional gene loci (chromosomal distribution shown in Fig. 3F, G) and optimized 7321 sites (Table S3).

**Fig. 4:**
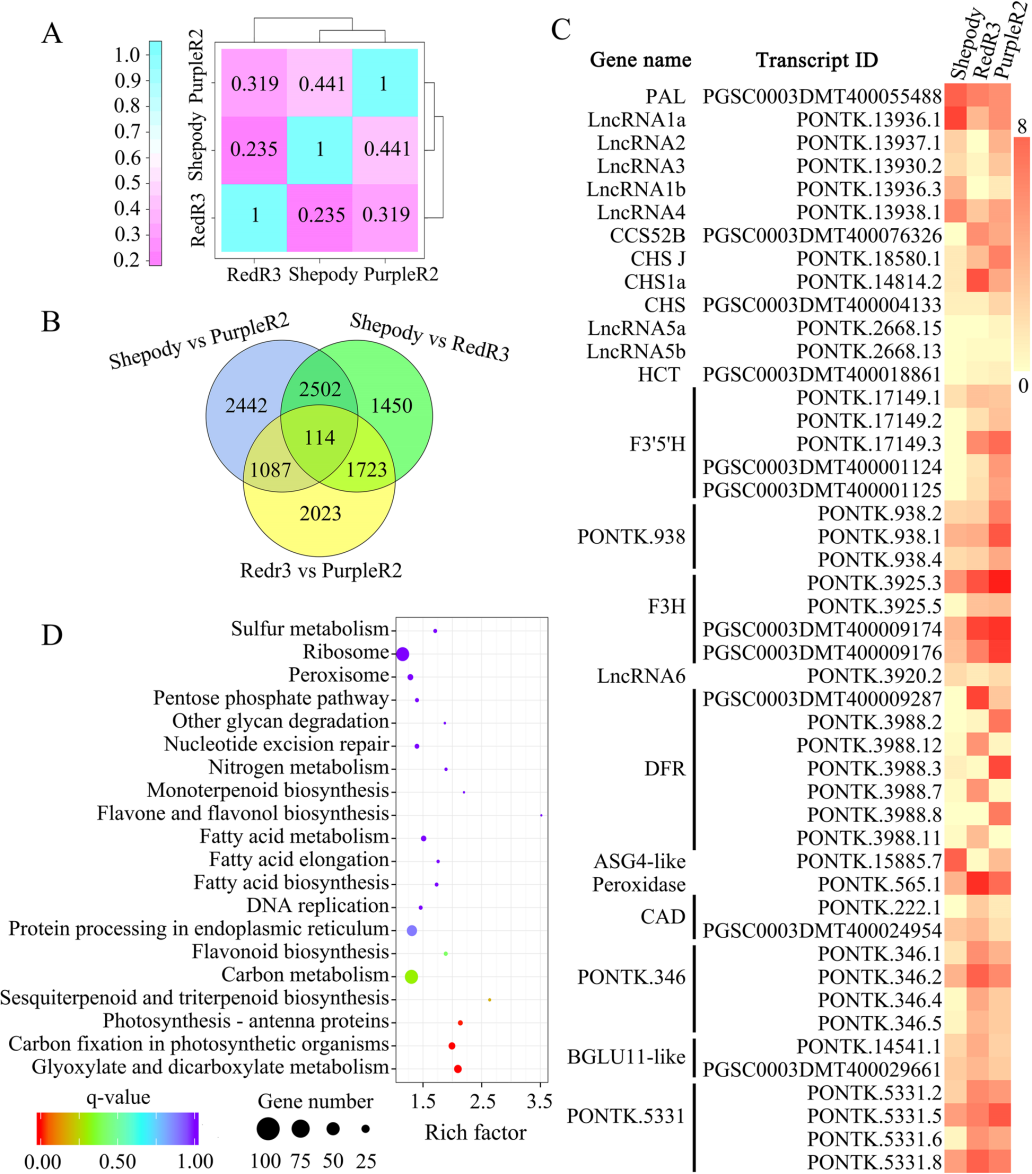
Differential expression of genes and KEGG enrichment in RedR3 and PurpleR2. **A**: Correlation between gene expression and color change in potato leaves of different colors. **B**: Statistics of DEG after comparing potato leaves of different colors. **C**: DEG related to anthocyanin synthesis and color change in potato leaves of different colors. **D**: KEGG enrichment analysis of differentially expressed transcripts in RedR3 and PurpleR2. (The enrichment factor represents the ratio of the proportion of genes annotated to a pathway in the differential genes to the proportion of genes annotated to the pathway in all genes. The color of the circle represents the qvalue, and the qvalue is the *P* value after multiple hypothesis test correction. The size of the circle indicates the number of genes enriched in the pathway)

From the full-length transcription sequencing data, we identified 1072 long noncoding RNA (lncRNA) transcripts (Table S4). Based on the reference genome annotation information for the genes on which these lncRNAs are located, they can be divided into four categories: large intergenic noncoding RNA (lincRNA), anti-sense lncRNA, intronic lncRNA, and sense lncRNA. Sense lncRNA includes gene promoter–related lncRNA and UTR-region lncRNA. Transcripts of lincRNA, sense lncRNA, anti-sense lncRNA, and intronic lncRNA were present in proportions of 60.4, 24.2, 14.2, and 1.2%, respectively (chromosomal lncRNA distribution shown in Fig. 3H–K). Gene annotation revealed that these lncRNAs regulate PAL, F3H, and CHS expression in the potato anthocyanin synthesis pathway (Figs. 3L, 4C). *PAL* was the target gene of lncRNA1a (PONTK.13936.1), lncRNA1b (PONTK.13936.3), lncRNA2 (PONTK.13937.1), lncRNA3 (PONTK.13930.2), lncRNA4 (PONTK.13938.1); *F3H* was the target of lncRNA6 (PONTK.3920.2) Gene; *CHS* was the target gene of lncRNA5a (PONTK.2668.13), lncRNA5b (PONTK.2668.15). LncRNA1a and lncRNA1b belong to anti-sense lncRNA; lncRNA2, lncRNA3, lncRNA4, lncRNA6 belong to lincRNA; lncRNA5a and lncRNA5b belong to sense lncRNA.

### Differential gene expression

The full-length transcriptome sequencing results were analyzed using Gene Ontology (GO) annotation and Kyoto Encyclopedia of Genes and Genomes (KEGG) enrichment. Gene expression was highly correlated with leaf color for Shepody and PurpleR2 (Pearson correlation coefficient, 0.441) but not for Shepody and RedR3 (Pearson correlation coefficient, 0.235) (Fig. 4A). These findings indicate that PurpleR2 and Shepody have more DEGs than RedR3 and Shepody. In summary, the number of anthocyanin synthesis–related DEGs was positively correlated with changes in leaf color from light to dark.

We then compared the transcript expression of the varieties in pairs (Fig. 4B). In total, 114 transcripts were differentially expressed among the varieties. These differentially expressed transcripts have important functions in regulating potato anthocyanin biosynthesis and color. Based on KEGG enrichment analysis of the significantly differentially expressed transcripts from the RedR3 and PurpleR2 leaves, many of the DEGs were enriched in the flavonoid biosynthetic pathway (KEGG pathway ko00941) (Fig. 4D). This indicates that differential gene expression in this pathway is an important driver of potato leaf color. Figure 4C shows the expression of significant DEGs related to potato anthocyanin biosynthesis and color differences; these include three forms of DFRa (PGSC0003DMT400009287, PONTK.3988.2, and PONTK.3988.12) and four of DFRb (PONTK.3988.3, PONTK.3988.7, PONTK.3988.8, and PONTK.3988.11). Relative to that in Shepody, DFR transcript expression was significantly upregulated in RedR3 and PurpleR2.

The transcript expression of the three transcriptome sequencing materials was compared in pairs (Fig. 4B). It can be seen that 114 transcripts were differentially expressed in the three potato varieties, and these transcripts have important functions in regulating potato anthocyanin biosynthesis and color changes. In order to study further, KEGG enrichment analysis was performed on the significantly differentially expressed transcripts in leaves of RedR3 and PurpleR2 (Fig. 4D). The results showed that a large number of DEG were enriched in the flavonoid biosynthetic pathway (ko00941). This indicates that the differential expression of genes in the flavonoid biosynthetic pathway is an important reason for the different colors of potato leaves. Based on the above results, the expression of significant DEG related to potato anthocyanin biosynthesis and color changes in potato leaves is shown in Fig. 4C. For the *DFR*, PGSC0003DMT400009287, PONTK.3988.2, and PONTK.3988.12 belong to the *DFRa* type; PONTK.3988.3, PONTK.3988.7, PONTK.3988.8, and PONTK.3988.11 belong to the *DFRb* type. The expression levels of *DFR* transcripts were lower in Shepody, but the expression of *DFR* was significantly up-regulated in RedR3 and PurpleR2.

### Combined transcriptome and metabolomic analysis

**Fig. 5:**
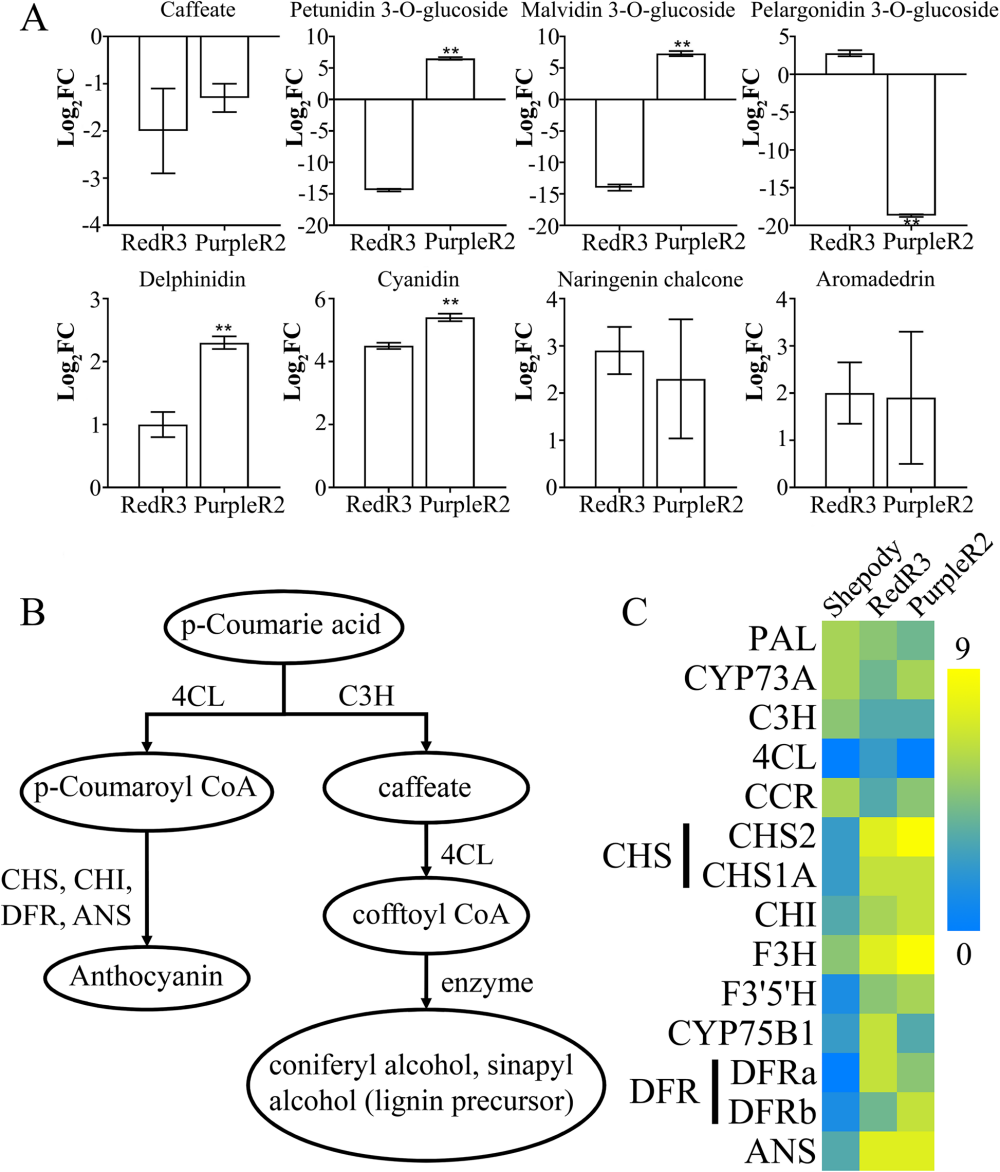
The difference between anthocyanins and key genes in potato leaves of different colors. **A**: Differences in the types and content of anthocyanins in potato leaves of different colors. **B**: Correlation analysis of differentially expressed genes and differential metabolites. **C**: Differences in the expression of key regulatory genes for phenylpropanoid and flavonoid metabolism in potato leaves of different colors

Figure 5A lists some of the anthocyanin-related metabolites after data quality screening. Compared with those in Shepody, naringenin chalcone and aromadendrin contents were significantly increased in the colored varieties, with cyanidin and delphinidin contents increasing more significantly in PurpleR2; petunidin 3-O-glucoside and malvidin 3-O-glucoside contents were significantly increased in PurpleR2 but significantly decreased in RedR3. In the phenylpropanoid synthesis pathway, coumaric acid is catalyzed by a series of enzymes to generate both lignin and anthocyanins (Fig. 5B). However, in the colored varieties, the expressions of C3H, CCR, and other enzymes in the lignin synthesis pathway were downregulated (Fig. 5C), as was caffeic acid expression, thereby limiting the production of the lignin precursors coumarin, coniferyl alcohol, and sinapal. In contrast, in the colored varieties, the expressions of genes related to the production of CHS, CHI, DFR, ANS, and other enzymes in the anthocyanin synthesis pathway were upregulated, and their anthocyanin content was higher. These findings indicate that gene upregulation in the flavonoid metabolic pathway has a key role in promoting anthocyanin accumulation and in producing color differences.

Relative to that in Shepody, RedR3 contained more cyanidin and pelargonidin 3-O-glucoside, and PurpleR2 contained more cyanidin, delphinidin, petunidin 3-O-glucoside, and malvidin 3-O-glucoside. Delphinidin, which accumulates in the form of glycosides, is the key reason for the red/purple color difference. This indicates that anthocyanin biosynthesis regulation occurs mostly downstream of anthocyanin synthesis during, for instance, flavonoid biosynthesis (ko00941).

### Transcriptomic data verification via quantitative reverse-transcription polymerase chain reaction (qRT-PCR)

**Fig. 6:**
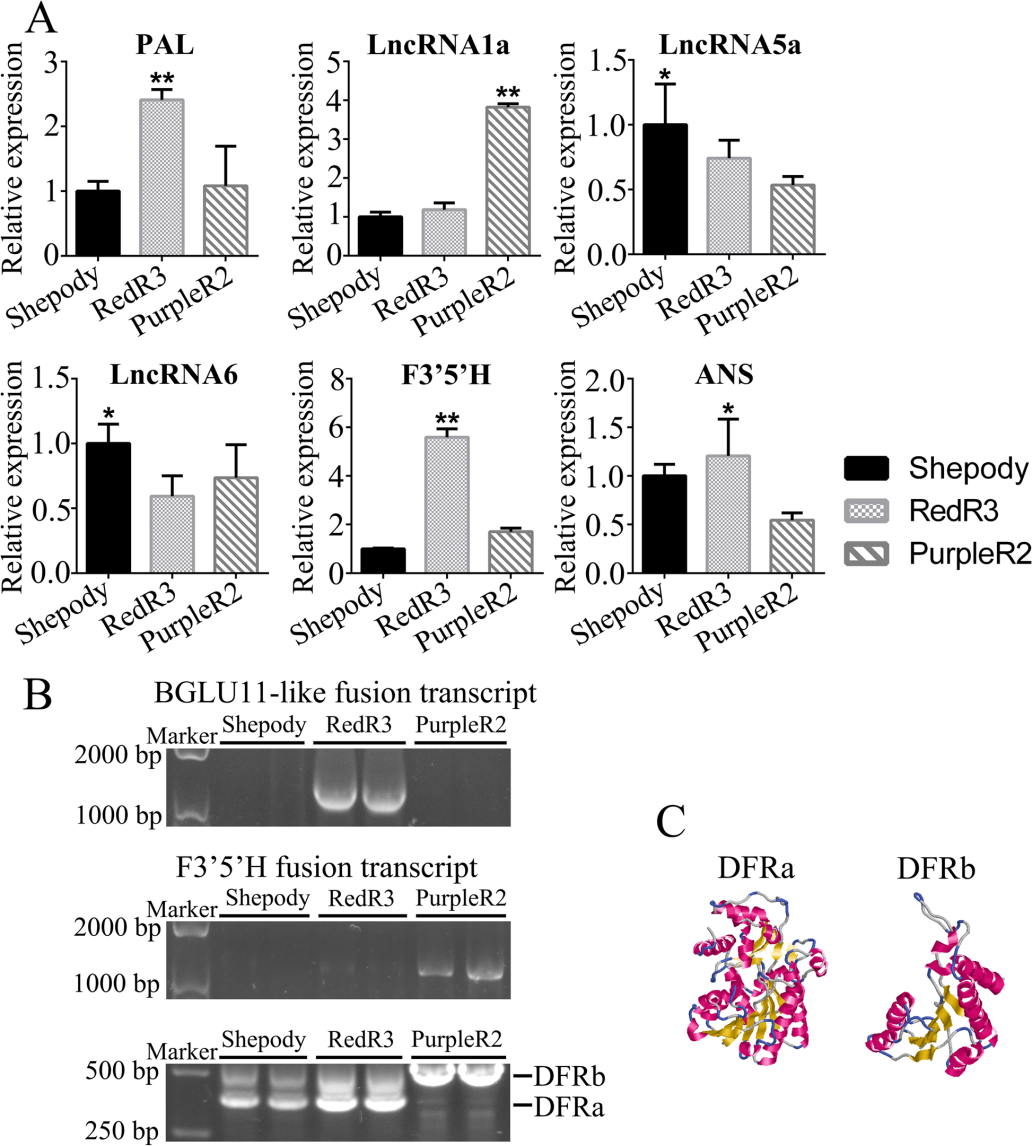
Verification of transcriptome anthocyanin related gene expressions. **A**: Expression of different transcripts in different potato leaves. **B**: Alternative splicing and fusion transcripts. **C**: Protein structure prediction of differential dihydroflavonol reductase transcription

We used qRT-PCR to verify the transcriptomic regulation of anthocyanin synthesis revealed via full-length transcriptome sequencing. For the six selected lncRNAs and key functional gene transcripts, PAL, lncRNA1a, lncRNA5a, lncRNA6, F3′5′H, and ANS, the qRT-PCR and RNA-seq results were consistent (Figs. 4C, 6A). RedR3 and PurpleR2 had opposite expression patterns for PAL and lncRNA1a. LncRNqA may negatively regulate PAL expression in colored varieties, F3′5′H gene expression was significantly upregulated (by 5.59-fold) only in RedR3.

The analysis results (Supplementary Fig. S6) for BGLU11-like fusion transcript expression in RedR3 were consistent with those of the transcriptome RNA-seq analysis (Fig. 6B). The F3′5′H fusion transcript was expressed in PurpleR2, was absent from Shepody (Fig. 6B), and was expressed at extremely low levels in RedR3 (Fig. 6B). Based on the gray value of the target band, F3′5′H fusion transcript expression was 8.57 times greater in PurpleR2 than that in RedR3. This indicates that F3′5′H plays a key role in anthocyanin synthesis and accumulation in the colored varieties but more so in PurpleR2 than in RedR3.

To verify DFR alternative splicing using primers on both sides of the DFR transcript intron-insertion site. We refer to the original annotated transcript without alternative splicing as DFRa; the alternatively-spliced transcript (hereafter DFRb) retains a 105 bp intron sequence between exons 3 and 4 (Fig. 6C). qRT-PCR revealed that intron retention in DFRb caused its expression to differ from that of DFRa. In RedR3, DFRa expression was 1.67 times greater than that of DFRb, and the intron-preserving alternative splicing was less likely to occur. In PurpleR2, DFRa expression was almost undetectable, with DFRb being predominant. These qRT-PCR results validate the DFR alternative splicing revealed by the full-length transcriptome sequencing results.

### Anthocyanin 1 (AN1) cloning and overexpression

Based on GO annotation, 23 DEGs were found to be associated with DNA binding (GO:0003677). One of these, PGSC0003DMG400013965, on chromosome 10, is the R2R3-MYB transcription factor AN1, whose expression was significantly upregulated in the colored varieties. Software prediction revealed that in the anthocyanin synthesis pathway, the MYB regulatory element or binding site is present in the 2000 bp CDS upstream of PAL, C3H, 4CL, CHS, CHI, F3H, DFR, and ANS. Searching the Potato Genome Sequencing Consortium (PGSC) database (http://solanaceae.plantbiology.msu.edu/pgsc_download.shtml) revealed two existing annotated transcripts of this gene, PGSC0003DMT400036281 and PGSC0003DMT400036283. Using our nanopore full-length transcriptome sequencing results for sequence alignment, we identified an *AN1* transcript (hereafter *StAN1n*). Transcript PGSC0003DMT400036281 contains exons a and c, and PGSC0003DMT400036283 contains exons a and b. *StAN1n* contains all three exons, a, b, and c. Relative to the known AN1 transcript sequence, we observed alternative splicing of the 5′ end of the exon a of StAN1n (Fig. S5); this also affected its CDS. We therefore subsequently cloned this transcript for further analysis.

**Fig. 7:**
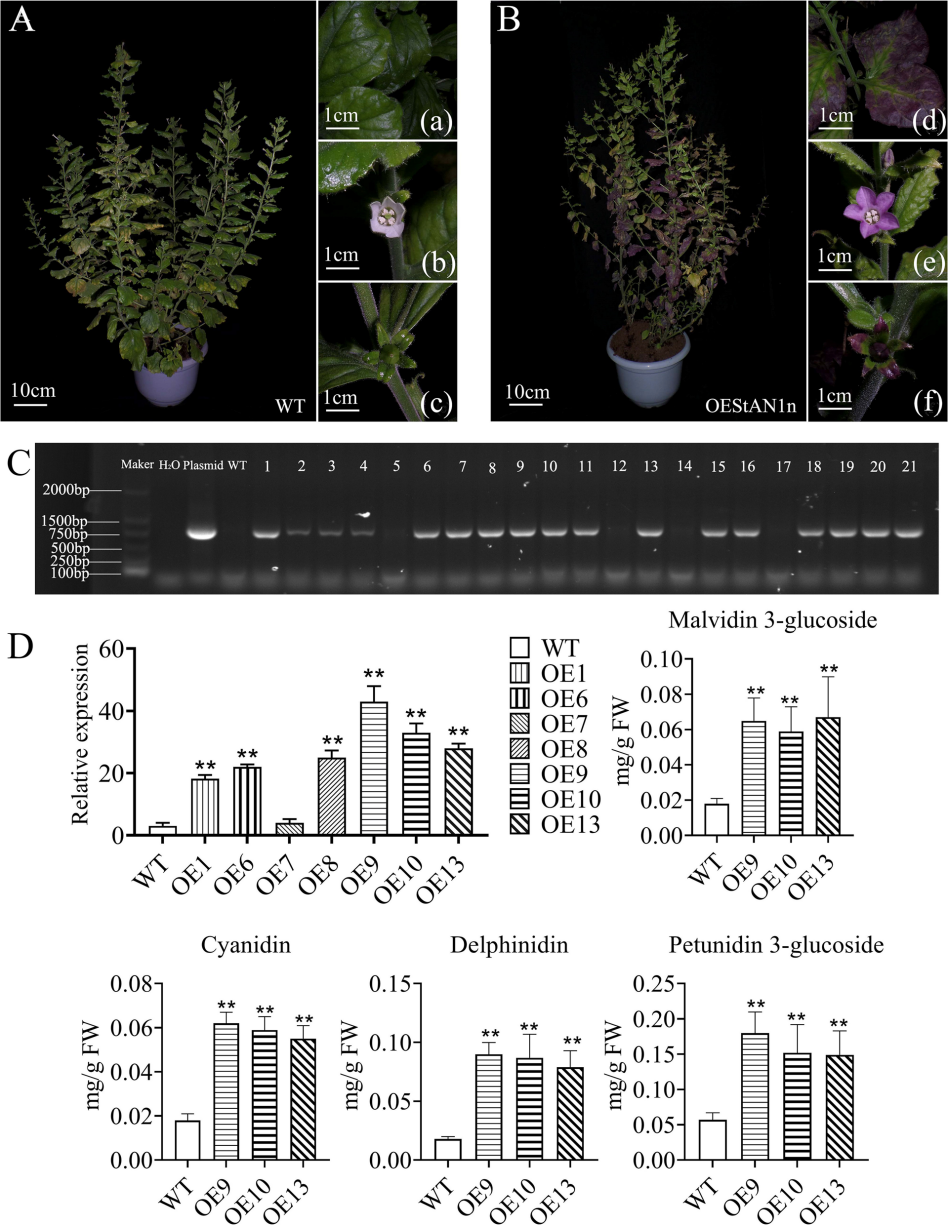
Identification and determination of anthocyanin content in transgenic tobacco. **A**: Wild-type tobacco. **B**: Genetically modified tobacco. **C**: Transgenic tobacco. **D**: Gene expression and anthocyanin content in transgenic tobacco

We then used qRT-PCR of the coding sequences corresponding to the *StAN1n* transcript in RedR3 and PurpleR2 to verify these results. Transgenic tobacco overexpressing *StAN1n* from the colored varieties (OEStAN1) was obtained via Agrobacterium transformation (Fig. 7A, B). After Agrobacterium transformation, the tobacco leaf callus color changed to purple. After strict selfing, the T2 transgenic tobacco StAN1n-positive rate was 81% (Supplementary Fig. S6). Using StAN1n-positive plants (Fig. 7C), we determined the anthocyanin content of plants with high *StAN1n* expression. Wild-type tobacco has white flowers, and green leaves and pods. OEstAN1 plants had purple leaves, flowers, and pods. These findings indicate that StAN1n plays an important role in regulating plant color.

We evaluated anthocyanin content in the WT and OEStAN1 tobacco leaves: it was lower in WT green leaves than in OEStAN1 leaves (Fig. 7D). This reveals that *StAN1n* overexpression promotes anthocyanin synthesis and accumulation in OEStAN1 transgenic tobacco, causing it to turn purple.

## Discussion

### Advancing potato genomics and transcriptomics

Whole-genome sequencing is essential for advancing potato-related molecular research. Nonetheless, published annotations of potato genome sequences [31] rely primarily on second-generation transcriptome sequencing data. Here, we utilized the longer read lengths and greater sequencing depths provided by third-generation sequencing to supplement and improve the published potato genome annotation data. Our in-depth mining of full-length transcriptome data elucidates the complex transcriptomic regulation of potato leaf color. Our findings reveal that potato color and anthocyanin accumulation and the type of anthocyanin produced are regulated by the differential expression of genes, transcriptomic lncRNAs, and fusion transcripts and by alternative splicing [32].

### Role of transcript fusion in anthocyanin biosynthesis

The function of anthocyanin biosynthesis–related genes is affected not only by their own expression [33] but also by the regulation of lncRNA interactions, gene transcript fusion, and alternative splicing [34, 35]. F3′5′H and PONTK.938 have undergone transcript fusion, and their expression patterns were similar, further indicating that they participate in the regulation of anthocyanin biosynthesis [36]. Our validation of the alternative splicing of DFR indicates that alternative splicing regulation affects anthocyanins synthesis. We were unable to verify the expression of the CAD and PONTK.346 fusion transcripts. Therefore, even when using nanopore full-length transcriptome sequencing, further analysis and experimental verification may be required.

### Anthocyanin accumulation regulation via the flavonoid biosynthesis pathway

**Fig. 8:**
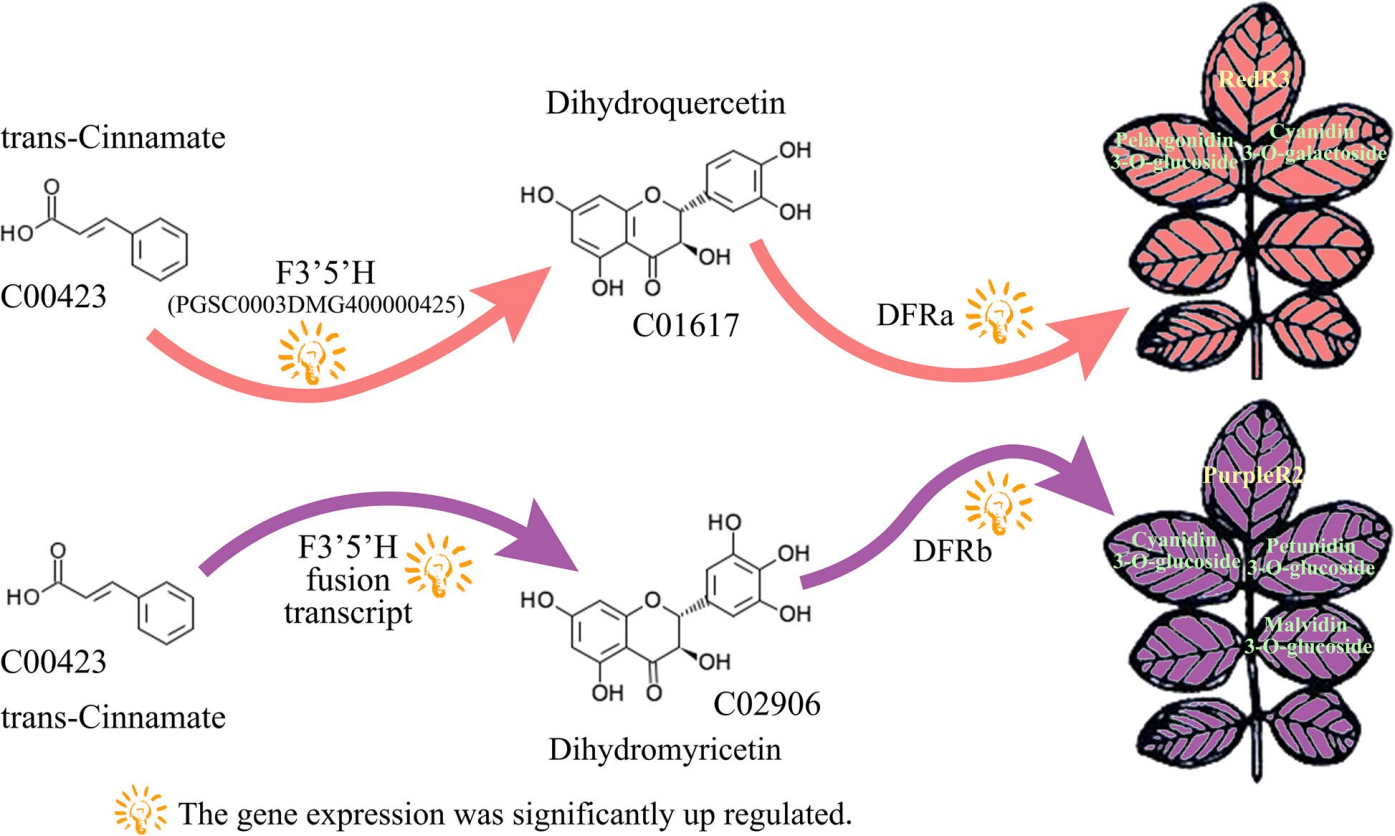
Differential expression proposition composed of differentially expressed genes and differential metabolites in RedR3 and PurpleR2 potato leaves

For p-coumaroyl-CoA entering the flavonoid biosynthesis pathway, the direction of metabolic transformation differed between the colored varieties. In the leaves of RedR3, the relative proportions of dihydrokaempferol, dihydroquercetin, and dihydromyricetin were 87.29, 1.38, and 11.24%, respectively; for PurpleR2, they were 81.44, 11.19, and 7.37% respectively. The combined proportions of dihydroquercetin and dihydromyricetin, precursors of cyanidin and delphinidin, respectively, the main anthocyanin species responsible for plant color, were 12.62% in RedR3 and 18.56% in PurpleR2. The conversion efficiency of dihydrokaempferol to cyanidin and delphinidin was at least 1.47 times greater in PurpleR2 than in RedR3. F3′5′H and CYP75B1 play important roles in the conversion of these metabolites [37]. In RedR3, the expressions of both F3′5′H and CYP75B1 were significantly upregulated, promoting the conversion of dihydrokaempferol to dihydroquercetin and thus cyanidin accumulation [38]. However, in RedR3, F3′5′H could not fuse with PONTK.938, thus limiting the conversion efficiency. In RedR3, dihydrokaempferol was not converted into dihydroquercetin and dihydromyricetin in large amounts. F3′5′H and PONTK.938 transcript fusion occurred in PurpleR2 (Fig. 8). Although this fusion promoted the conversion of dihydrokaempferol to dihydroquercetin and dihydromyricetin, it almost eliminated the conversion of naringenin into eriodictyol in the leaves of PurpleR2, causing eriodictyol to be almost undetectable. In PurpleR2, this fusion promoted cyanidin and delphinidin accumulation [39].

The expression of the two alternatively-spliced DFR transcripts, DFRa and DFRb, varied between the colored varieties (Fig. 8) and was lower in Shepody, the control. DFRa expression was greater than DFRb expression in RedR3 but less than that in PurpleR2, consistent with the differences in cyanidin and delphinidin content between these varieties [40]: at the higher DFRa-type transcript spliceosomes content, cyanidin 3-O-galactoside, pelargonin, and pelargonidin 3-O-glucoside accumulated, producing the red color [41], and at the higher DFRb-type content, petunidin 3-O-glucoside, malvidin 3-O-glucoside, delphinidin 3-O-glucoside, and cyanidin 3-O-glucoside accumulated, producing the purple color [42]. In tobacco, AN1 overexpression caused anthocyanin accumulation, leading to purple leaves. Together, these findings indicate that anthocyanin accumulation in plants is regulated by transcription factors, genes, and processing during transcription.

KEGG enrichment analysis of RNA-seq–derived DEGs identified metabolic pathways other than the flavonoid biosynthetic pathway (ko00941) that may also affect color formation in potato leaves. The significantly enriched pathways include “sesquiterpenoid and triterpenoid biosynthesis” (ko00909), “photosynthesis-antenna protein” (ko00196), “carbon fixation in photosynthetic organisms” (ko00710), and “glyoxylate and dicarboxylate metabolism” (ko00630) [43, 44]. Although our findings have elucidated these mechanisms, color formation in plant leaves is a complex process, and the effects of these pathways on potato leaf color require further in-depth analysis.

## Conclusions

By applying extensive targeted metabolomics and nanopore full-length transcriptome analysis to elucidate the anthocyanin synthesis pathway, we detected 17 anthocyanins. The expressions of most of the structural genes in this pathway were upregulated in the colored varieties, increasing their anthocyanin content. The leaves of PurpleR2 had higher petunidin 3-O-glucoside and malvidin 3-O-glucoside content, and those of RedR3 had higher pelargonidin 3-O-glucoside content. We identified 114 significantly DEGs. Transcription factors in multiple families were detected, the most abundant being in the C3H family, followed by those of the MYB family. We therefore overexpressed an MYB transcription factor, StAN1n, in tobacco, finding that it promoted anthocyanin accumulation, causing the tobacco leaves to turn purple. These findings elucidate anthocyanin synthesis and regulation and their association with leaf color in potato leaves.

## Methods

### Plant materials

The potato Shepody with green leaves and white tubers was used as control. The Red Rose3 (RedR3) potato variety with red leaves, tubers, and skins and Purple Rose 2 (PurpleR2) with purple leaves, tubers, and skins were used as test materials (The Northwest Agriculture and Forestry University, Yangling, China, provided the experimental plant materials). The potato seed tubers were planted in a greenhouse and subjected to 16 h of light and 8 of darkness at 22 °C. Potato leaves were sampled 45 days after emergence and immediately frozen in liquid nitrogen until the extraction of total RNA and total metabolites. All experiments were replicated thrice.

### Measurement of anthocyanin content

First, potato leaves were ground using a mortar, and 1 mL of 70% ethanol was added. Next, the ground tissues were centrifuged at 12,000 g at 4 °C for 15 min. Next, 500 μL of the supernatant was extracted, and 1.5 mL was added to pH 1.0 and 4.5 buffer solution, respectively, and balanced at 40 °C for 30 min. Next, the absorbance was measured using an ultraviolet spectrophotometer for the two buffers at a wavelengths of 525 and 700 nm [45, 46], and ethanol was used as blank. The analysis of each sample was replicated thrice.

### Metabolite extraction

First, the freeze-dried leaves were crushed at 30 Hz for 15 min using a mixer mill (MM 400, Retsch, Haan, Germany) with a zirconia bead. Next, 100 mg of the leaf powder was mixed with 1.0 ml of 70% aqueous methanol and incubated overnight at 4 °C for metabolite extraction. Next, the extracts were centrifuged at 10,000 g for 10 min, absorbed using a Carbon-GCB SPE Cartridge (ANPEL, Shanghai, China), and filtrated using SCAA-104 filter (ANPEL) before liquid chromatography-mass spectrometry analysis.

### Ultra-performance liquid chromatography (UPLC) analysis

The sample extracts were analyzed using an liquid chromatography-electrospray ionization-mass spectrometry system (Shimadzu, Kyoto, Japan). The analytical conditions were as follows high performance-liquid chromatography: column, Waters (1.8 μm, 2.1 mm *100 mm); solvent system, water (0.04% acetic acid); acetonitrile (0.04% acetic acid); gradient program, 95:5 V/V at 0 min, 5:95 V/V at 11.0 min, 5:95 V/V at 12.0 min, 95:5 V/V at 12.1 min, 95:5 V/V at 15.0 min; flow rate, 0.40 ml/min; temperature, 40 °C; injection volume: 2 μL. The effluent was connected to an ESI-triple quadrupole-linear ion trap (Q TRAP)-MS.

### MS/MS conditions

The LIT and triple quadrupole (QQQ) scans were obtained using a triple quadrupole-linear ion trap mass spectrometer (Q TRAP) (Sciex, Framingham, MA, USA), API 6500 Q TRAP LC/MS/MS System (Sciex), equipped with an ESI Turbo Ion-Spray interface (Sciex), operating in a positive ion mode and controlled using the analyst 1.6.3 software (Sciex). The ESI source operation parameters were as follows: ion source, turbo spray; source temperature, 500 °C; ion spray voltage, (IS) 5500 V; ion source gas I (GSI), gas II (GSII), and curtain gas (CUR) were set at 55, 60, and 25.0 psi, respectively; the collision gas (CAD) was high. Instrument tuning and mass calibration were performed with 10 and 100 μmol/L polypropylene glycol solutions in QQQ and LIT modes. QQQ scans were acquired as multiple reaction monitoring (MRM) experiments with collision gas (nitrogen) set at 5 psi. The declustering potential (DP) and collision energy (CE) for individual MRM transitions were done with further DP and CE optimization. A specific set of MRM transitions were monitored for each period according to the metabolites eluted within the period.

### Identification and quantitative analysis of different metabolites

The analyst 1.6.3 software (Sciex) was used to read and process the mass spectrum data. Qualitative and quantitative analysis of the metabolites of the samples were conducted using mass spectrometry based on the Human Metabolome Database (https://hmdb.ca/), MetaboLights (https://www.ebi.ac.uk/metabolights/), Golm Metabolome Database (http://gmd.mpimp-golm.mpg.de/), and the local metabolic metware database (MWDB) provided by BioMarker technologies, Rohnert, CA, USA. The characteristic ion of each substance was screened out using the triple quadrupole for LC/MS, and the signal intensity of the characteristic ion was obtained in the detector. The mass spectrum file of the sample was opened using the MultiaQuant 3.0.2 software (Sciex) to integrate and correct the chromatographic peak. The area under each chromatographic peak represents the relative content of the corresponding metabolite. All chromatographic peak area data were exported for further analysis.

Principal component analysis (PCA) was used to establish a mathematical model to summarize the metabolome analysis results of colored potato leaves. Orthogonal partial least squares discriminant analysis (OPLS-DA) was used to construct the OPLS-DA model based on the metabolome results, and the arrangement of the constructed model was verified (*n* = 200). The multivariate analysis OPLS-DA model calculated the variable importance in project (VIP) values. The screening criteria for differential metabolites were metabolites with products that differed by more than two or less than 0.5 between the control and the experimental group and VIP ≥ 1. In addition, by searching the Kyoto encyclopedia of genes and genomes (KEGG) database [47], metabolomics products with significantly different contents were metabolic pathways obtained through enrichment analysis.

### RNA extraction and nanopore sequencing

Potato leaf samples were frozen with liquid nitrogen, and the full-length transcriptome was sequenced using nanopore technology. The pure plant total RNA extraction kit (DP441, TIANGEN, Tianjin, China) extracted total RNA. The Qubit Fluorometer and NanoDrop 2000 (Thermo Fisher, Waltham, MA, USA) were used to detect the concentration and purity of total RNA samples. The OD260/280 values of extracted total RNA from potato leaves ranged from 2.0 to 2.2. Agilent 2100 (Agilent Technologies, Wilmington, DE, USA) was used to detect 28S/18S and RIN values of total RNA samples. We used VAHTS mRNA capture beads (Vazyme, Nanjing, China) to enrich and purify the RNA with Poly (A) + tail from 1 μg of total RNA. In this study, 1 ng Poly (A) + RNA was used. The cDNA-PCR Sequencing Kit (Oxford Nanopore Technologies, UK) and PCR Barcoding Kit (Oxford Nanopore Technologies) were used to synthesize double-stranded cDNA by PCR. We used the NEBNext FFPE DNA Repair Mix (New England Biolabs, Ipswich, MA, USA) and NEBNext Ultra II End Repair/dA-Tailing Module (New England Biolabs) to repair damaged nucleic acid fragments, and the end repair plus A. Finally, the Rapid Adapter (RAP) in the cDNA-PCR Sequencing Kit (Oxford Nanopore Technologies SQK-PCS109, UK) connected the sequencing adapter and constructed the cDNA library required for sequencing. The PromethION flow cells (Oxford Nanopore Technologies) were used to construct cDNA library sequenced on the PromethlON 48 platform. The analysis of each sample included three biological replicates.

### RNA-seq data analysis and annotation

In this study, the EBSeq software [48] was used for gene differential expression analysis. For detecting differentially expressed genes, log2 (Fold Change) ≥ 2 and FDR < 0.01 were used as screening criteria. The DEG obtained were compared in NCBI non-redundant protein sequences (NR) in Gene Ontology (GO) to obtain annotation information. The GOseq software [49] was used for GO enrichment, and KOBAS [50] was used to KEGG annotate. We used the topGO, ggplot2, and circos 0.69 to visualize the results. The cDNA_cupcake software analyzed the fusion transcripts. The sequence obtained by sequencing the full-length transcriptome before the removal of redundancy was screened to identify the fusion transcripts in each sample. The criteria and principles for fusion candidates were that a single transcript must meet the following conditions simultaneously [51, 52]. (a) It must map to 2 or more loci. (b) The minimum coverage for each loci should be 5%, and the minimum coverage should be greater or equal to 1 bp. (c) Total coverage should be greater or equal to 95%. (d) Distance between the loci should be at least 10 kb. The transcript sequence was obtained by sequencing the full-length transcriptome after removal of redundancy was compared with the transcript sequence of the known gene of potatoes using the gffcompare software. The original genome annotation information was supplemented and improved.

### Verification of anthocyanin biosynthesis gene expression using the qRT-PCR

200 mg potato leaves of each sample were quickly frozen with liquid nitrogen, and the RNAprep Pure plant total RNA extraction kit DP441 (TIANGEN) was used to extract total RNA. Next, the TIANScript II cDNA first strand synthesis Kit (TIANGEN) synthesized cDNA. The qRT-PCR experiments were performed with the EF-1α gene as a reference. The SuperReal PreMix Color (SYBR Green) kit (TIANGEN) was used for qRT-PCR using the QuantStudio 7 Flex Real-Time PCR System (Thermo Fisher). The reaction conditions were: 95 °C for 15 min; 95 °C for 10 s, 60 °C for 20 s, 72 °C for 20 s plate Read; 40 cycles were performed, and the melting curve was drawn. The 2–ΔΔCt method was used to calculate the relative expression of each gene. The primers used for qRT-PCR are shown in Table S7. The online tool Phyre2 was used to predict the protein tertiary spatial structure of DFRa type transcript PGSC0003DMT400009287 and DFRb type transcript PONTK.3988.8 proteins. The analysis of each sample included three biological replicates.

### Cloning of the AN1 gene and transformation of tobacco

RT-PCR was used to clone the *StAN1n* transcript in Shepody, RedRose3(RedR3), and PurpleRose2 (PurpleR2). The primers used for cloning are presented in Supplementary Table S7. KOD-Plus-Neo was used for PCR amplification. The annealing temperature of the PCR reaction was 51 °C, and the length of the target fragment was 798 bp in RedR3. The CaMV35S was used as the promoter of *StAN1n*. The plant expression vector with overexpressed *StAN1n* was constructed. The recombinant plasmid was transferred into Agrobacterium LBA4404. Tobacco (Nicotiana benthamiana) leaves were infected with Agrobacterium using the tobacco leaf transformation, and transgenic tobacco plants with StAN1n of RedR3 (OEStAN1n) overexpression were obtained.

### Identification of StAN1 transgenic tobacco

PCR detection of StAN1 transgenic tobacco: a DNA extraction kit was used to extract the DNA of T4 transgenic tobacco with the StAN1 gene. PCR amplification was accomplished with identification primers, reaction system 50 μL, DNA template 2 μL, upstream and downstream primers 2.5 μL each (10 mmol/L), ddH2O 18 μL, 2 × Taq PCR StarMix 25 μL, reaction program: 94 °C pre-denaturation 7 min; denaturation at 94 °C for 30 s; annealing at 51 °C for 30 s; extension at 72 °C for 1 min, 36 cycles; extension at 72 °C for 10 min, and storage at 4 °C.

QRT-PCR detection of transgenic tobacco with StAN1 gene: the total RNA of tobacco with positive PCR results were extracted, reverse transcription was done according to Tiangen Reverse Transcription Kit manufacturer guidelines, and the real-time fluorescence quantitative detection with a 20 μL system was performed according to the instructions of Tiangen Fluorescence Quantitative Kit manufacturer.

### Statistical analysis

Statistical analysis was performed using Excel 2016 software (Microsoft Office, USA). Revelant experiments were repeated 3 times. Data are presented as SD. The leavels of statistical significance were analyzed by the least significant difference(*p* < 0.05).

## Supplementary Information

**Additional file 1: Supplemental Table S1.** Differential metabolites in different potatoes.

**Additional file 2: Supplemental Table S2.** Different expression transcripts.

**Additional file 3: Supplemental Table S3.** Gene annotation information.

**Additional file 4: Supplemental Table S4.** LncRNA transcripts.

**Additional file 5: Supplemental Fig. S5** Sequence alignment of StAN1.

**Additional file 6: Supplemental Fig. S6** Original gels.

**Additional file 7: Supplemental Table S7.** Primers for PCR.

## Data Availability

The raw sequence data reported in this paper have been deposited in the Genome Sequence Archive (Genomics, Proteomics & Bioinformatics 2017) in National Genomics Data Center (Nucleic Acids Res 2020), Beijing Institute of Genomics (China National Center for Bioinformation), Chinese Academy of Sciences, under accession number CRA003703 that are publicly accessible at https://bigd.big.ac.cn/gsa. Regarding transcriptomics data analysis, with the reference genome DM_v4.04_pseudomolecules.fasta.

## Abbreviations

MBW complexes: MYB bHLH and WD40
PAL: Phenylalanine ammonia lyase
4CL: 4-coumarate-CoA ligase
CHS: Chalcone synthase
CHI: Chalcone isomerase
F3’5’H: Flavonoid 3′,5′-hydroxylase
DFR: Dihydroflavonol 4-reductase
ANS: Anthocyanidin synthase
lincRNA: Large intergenic noncoding RNA
lncRNA: Long non-coding RNA
KEGG: Kyoto Encyclopedia of Genes and Genomes
PGSC: The Potato Genome Sequencing Consortium
CDS: Coding DNA Sequence
UPLC: Ultra-performance liquid chromatography
CAD: Collision-activated dissociation
QQQ: Triple quadrupole
DP: Declustering potential
CE: Collision energy
PCA: Principal component analysis
OPLS-DA: Orthogonal partial least squares discriminant analysis
VIP: Variable importance in project
DEG: Differentially expressed genes
GO: Gene Ontology
qRT-PCR: Quantitative real-time polymerase chain reaction
