# Supplementary material for: Anthocyanin regulatory networks in Solanum tuberosum L. leaves elucidated via integrated metabolomics, transcriptomics, and StAN1 overexpression
Source: BMC Plant Biol. 2022 May 4;22:228. doi: 10.1186/s12870-022-03557-1 (PMC9066749; doi:10.1186/s12870-022-03557-1)
Supplement: Supplementary file 6 — Additional file 6: Supplemental Fig. S6 Original gels. [file 12870_2022_3557_MOESM6_ESM.docx]

Figure S6 Original gels

Gels of figure 6B


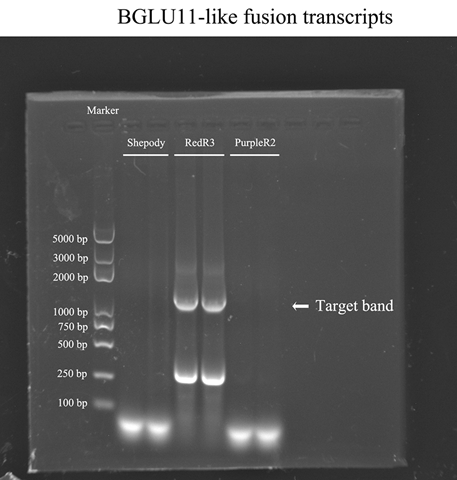

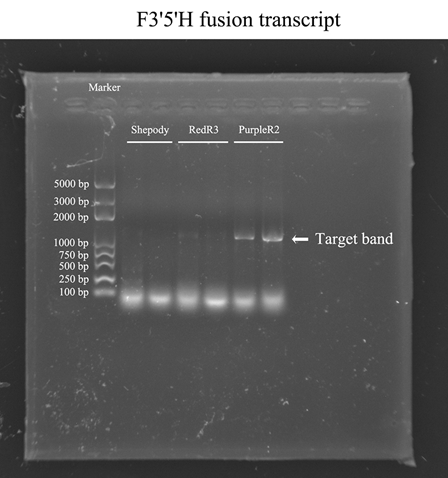


The RT-PCR method was used to verify the fusion transcript prediction results in the transcriptome sequencing. It was the original gel image of agarose gel electrophoresis of the fusion transcript verification result. The RT-PCR experiment verified the original gel image of agarose gel electrophoresis of *BGLU11-like* fusion transcript in the transcriptome sequencing result. The RT-PCR experiment verified the original gel image of agarose gel electrophoresis of *F3’5’H* fusion transcript in the transcriptome sequencing result.


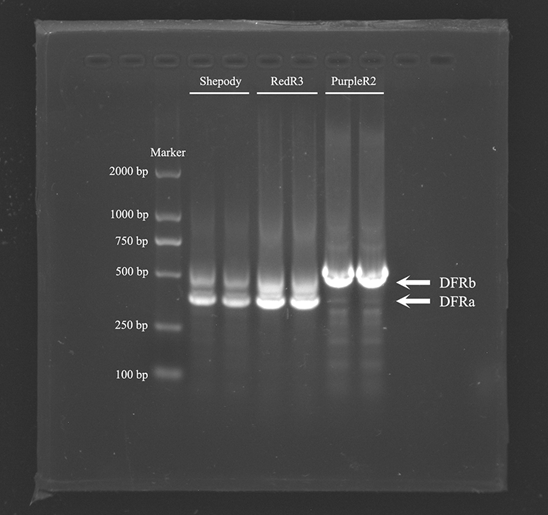


The RT-PCR was used to verify the alternative splicing of *DFR* gene in the potato leaves of different colors in the transcriptome sequencing results. It was the original gel image of agarose gel electrophoresis showing the alternative splicing of *DFR* gene in potato leaves of different colors.

Gel of figure7C


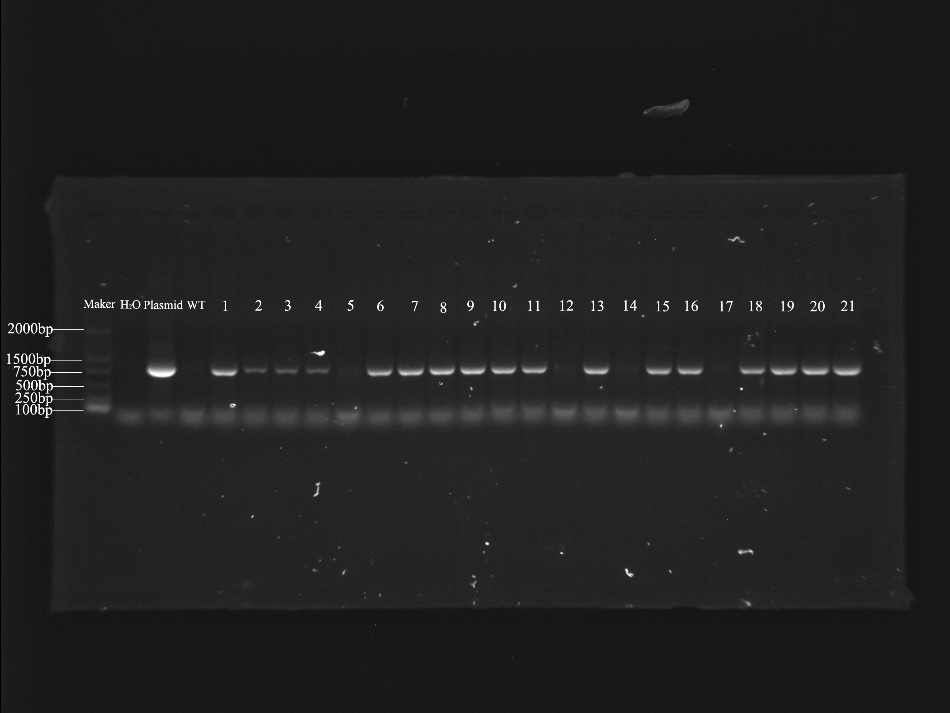


The PCR method was used to identify the transgenic plants. It was the original gel image of agarose gel electrophoresis .1-21means transgenic plants.
